# 

**Published:** 1849-11

**Authors:** 


WHOLE SERIES—VOL. VI, NO. IV.

VOL. II.] NOVEMBER, 1849. [No. 4.

Dr Luther Jewett

57

### THE NORTH-WESTERN

### MEDICAL AND SURGICAL

### JOURNAL.

EDITED BY

JOHN EVANS, M.D.,

PROFESSOR OF OBSTETRICS ETC., IN RUSH MEDICAL COLLEGE.

AND

EDWIN G. MEEK, M.D

PUBLISHED EVERY OTHER MOTH.

AT TWO DOLLARS PER ANNUM IN ADVANCE.

CHICAGO AND INDIANAPOLIS:

C. A. SWAN, PRINTER.

1849.

ENLARGED SERIES—VOL. IV, NO. IV.

